# DNA methylation differences in monozygotic twin pairs discordant for schizophrenia identifies psychosis related genes and networks

**DOI:** 10.1186/s12920-015-0093-1

**Published:** 2015-05-06

**Authors:** Christina A Castellani, Benjamin I Laufer, Melkaye G Melka, Eric J Diehl, Richard L O’Reilly, Shiva M Singh

**Affiliations:** Department of Biology, The University of Western Ontario, N6A 5B7 London, Ontario Canada; Department of Psychiatry, The University of Western Ontario, N6A 5B7 London, Ontario Canada

**Keywords:** Monozygotic Twins, Schizophrenia, DNA Methylation, MeDIP, Methylation Array, Differentially Methylated Regions (DMRs), snoRNA, Histone Clusters

## Abstract

**Background:**

Despite their singular origin, monozygotic twin pairs often display discordance for complex disorders including schizophrenia. It is a common (1%) and often familial disease with a discordance rate of ~50% in monozygotic twins. This high discordance is often explained by the role of yet unknown environmental, random, and epigenetic factors. The involvement of DNA methylation in this disease appears logical, but remains to be established.

**Methods:**

We have used blood DNA from two pairs of monozygotic twins discordant for schizophrenia and their parents in order to assess genome-wide methylation using a NimbleGen Methylation Promoter Microarray.

**Results:**

The genome-wide results show that differentially methylated regions (DMRs) exist between members representing discordant monozygotic twins. Some DMRs are shared with parent(s) and others appear to be *de novo*. We found twenty-seven genes affected by DMR changes that were shared in the affected member of two discordant monozygotic pairs from unrelated families. Interestingly, the genes affected by pair specific DMRs share specific networks. Specifically, this study has identified two networks; “cell death and survival” and a “cellular movement and immune cell trafficking”. These two networks and the genes affected have been previously implicated in the aetiology of schizophrenia.

**Conclusions:**

The results are compatible with the suggestion that DNA methylation may contribute to the discordance of monozygotic twins for schizophrenia. Also, this may be accomplished by the direct effect of gene specific methylation changes on specific biological networks rather than individual genes. It supports the extensive genetic, epigenetic and phenotypic heterogeneity implicated in schizophrenia.

**Electronic supplementary material:**

The online version of this article (doi:10.1186/s12920-015-0093-1) contains supplementary material, which is available to authorized users.

## Background

Monozygotic twins (MZ) have long been used to ascertain the genetic and environmental contributions to complex diseases, including schizophrenia [1]. Their unique aptness lies in originating from the genetic content of a single zygote and sharing most *in utero* and postnatal environments. Historically, the concordance and discordance for a disease between MZ twins has been attributed to unspecified genetic and environmental factors, respectively [2]. The recent advent of comprehensive genetic and epigenetic technologies has added a new supremacy to such studies. Studies of this kind hold the potential to identify specific mechanisms that contribute to the causation of disease [3,4]. The first stage in these studies is to identify differences between monozygotic discordant (MZD) twins that are expected to be genetically identical. The results during the last few years have established that differences do exist at the genetic [5,6] as well as epigenetic [7-9] levels. The results argue that MZ twins are similar, but not identical [6]. Also, the rare *de novo* mutations may take place during developmental mitosis during ontogeny [6].

Interestingly, methylation differences between identical twins have been reported as early as in newborns [10]. DNA methylation is reported to increase with age [11] and accordingly, the methylation differences between monozygotic twins increases with age [7]. Epigenetic differences between MZ twins include features like X-inactivation, genomic imprinting, or differential methylation of genes, and may cause MZ twin pairs to diverge, leading to disease discordance [3,12]. Studies of this kind have concluded that no two individuals are alike; not even identical twins [6]. However, the genetic similarity between MZ twins is comparable to no other two individuals. In addition, identical twins are matched for age, sex, maternal environment, and population cohort effects - making them the best matched control available [13]. Indeed, MZ twins provide a unique backdrop to assess epigenetic states that are shared due to inheritance or common environments, as well as differences that may be in response to individual specific exposures or random events [7-9,14,15]. Such changes, if operational, may allow monozygotic twins to develop discordance for almost any trait through reprogramming of gene expression via epigenetic mechanisms which may increase liability to disease [16]. This is particularly relevant in neurodevelopmental disorders, especially schizophrenia, and reports are now accumulating from twin studies to support an epigenetic model of disease contribution. For instance, it has been shown that the schizophrenic twin from a pair of discordant twins is epigenetically more similar to the affected concordant twins than to his own unaffected co-twin at the *DRD2* gene [17]. In addition, methylation of genes in blood samples of twins discordant for schizophrenia, including medication free patients, shows hypermethylation and hypomethylation of several genes [18,19].

Indeed, the molecular results accumulating on schizophrenia are encouraging and include many recent reports of associations between DNA methylation and schizophrenia [12,20,21]. The evidence is also emerging for lncRNAs as an important epigenetic contributor to schizophrenia [22].

The research presented here identifies genes whose methylation is altered in schizophrenia patients as compared to their unaffected twin using genome-wide assessment by Methylated DNA Immunoprecipitation (MeDIP) on a Nimblegen Human DNA Methylation Microarrays. It uses blood DNA from two sets of monozygotic twin pairs discordant for schizophrenia and their parents. The results identify DNA methylation differences between MZD twins in two families discordant for schizophrenia. Also, the patients across families share affected genes, and more importantly, biological networks. The implications of the results will require reports on an increasing number of MZD twins but are particularly promising given that the genes and networks identified are similar to accumulating reports.

## Methods

**Figure 1:**
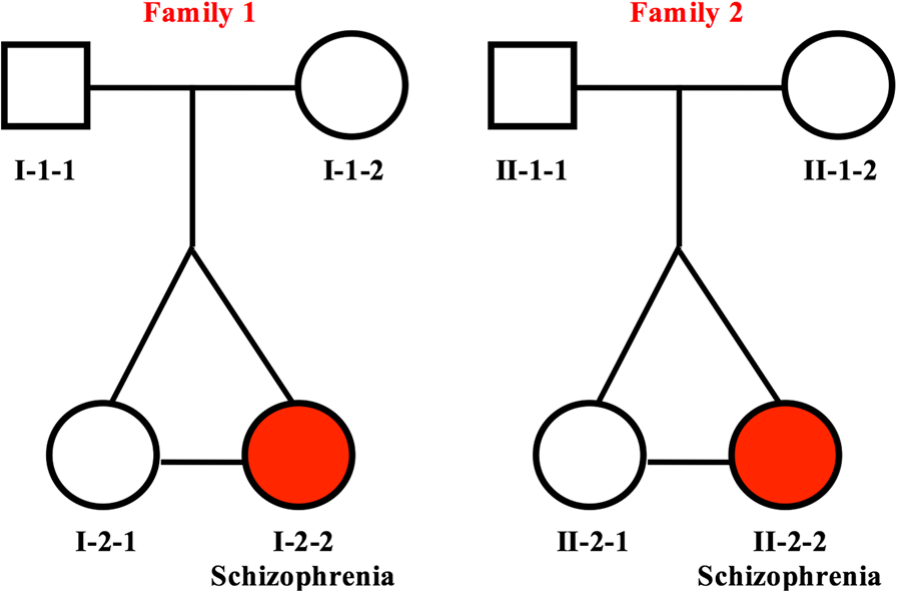
Pedigrees of families included in the study. Shaded circles represent the affected twins.

This study on monozygotic twins received ethics approval by the University of Western Ontario’s Committee on research involving human subjects. All subjects provided written informed consent to participate in this study. Further, they have agreed to the sharing of data (genetic and clinical) in any publication. All of the patients were adults at the time of consent. Capacity for consent was ensured using three measures 1) Schizophrenic patients gave consent only during a “normal” phase (no psychosis present), 2) Both twins of the twin pair were present and gave consent at the same time (the normal twin and their affected sibling), 3) If R.O’Reilly felt that capacity to consent was compromised, the patients were not included in our study. They were interviewed and clinically assessed by a single senior Psychiatrist (R. O’Reilly) using the SCID-I and SCID-II [23,24]. Past clinical notes were available to aid in diagnosis. Both families were comprised of identical female twins. The twins from Family 1 (Figure 1) were Caucasian females aged 43. The affected member of twin pair 1 was diagnosed with schizoaffective disorder at age 27. The twins were discordant for 16 years at the time of sample collection. The twins from Family 2 (Figure 1) were Afro-American females aged 53. The affected member of twin pair 2 was diagnosed with schizophrenia at age 22. The twins were discordant for 31 years at the time of sample collection. The twins and their parents (Figure 1) included in this study contributed whole blood samples for DNA isolation. Both pairs of monozygotic twins were female twins. DNA was extracted from whole blood using the 5 Prime Perfect Pure DNA Blood Kit (Gaithersburg, MD, USA), following the manufacturer’s protocol. It should be noted that the Father of Twin Pair 2 was diagnosed with Chronic Leukemia (CLL) at age 69. The affected patient from Family 1 was treated for schizophrenia symptoms using a combination of the medications Seroquel, Effexor and Topiramate. The affected patient from Family 2 was treated for schizophrenia symptoms using a combination of the medications Clozapine, Divalproex and Benztropine. Zygosity was confirmed by Affymetrix 6.0 microarray and specifically using the Affymetrix Genotyping Console 4.0 concordance feature [6].

The genomic DNA was processed at ArrayStar Inc (Rockville, MD, USA); this included the methylated DNA immunoprecipitation (MeDIP), sample labeling, and hybridization to the NimbleGen Human DNA Methylation Promoter Plus CpG Island 720k Array. The NimbleGen Human DNA Methylation 3x720k CpG Island Plus RefSeq Promoter Microarray is a multiplex slide with 3 identical arrays per slide. Each Roche Nimblegen Inc (Madison, MI, USA) array covers 27,728 annotated CpG islands as well as 22,532 promoters of the RefSeq genes derived from the UCSC RefFlat files. Median-centering, quantile normalization, and linear smoothing was performed by Bioconductor packages *Ringo*, *limma,* and *MEDME* at ArrayStar. Lastly, in order to compare the two groups’ differentially enriched regions the average of the log2-ratio values for each group (i.e. experimental patient [E] and healthy control [C]) was used to calculate M’ value (defined by the following equation) for each probe.

$$ \mathbf{M}' = \mathbf{Average}\left(\mathbf{log}\mathbf{2}\ \mathbf{MeDI}{\mathbf{P}}_{\mathbf{E}}/\mathbf{Inpu}{\mathbf{t}}_{\mathbf{E}}\right) - \mathbf{Average}\left(\mathbf{log}\mathbf{2}\ \mathbf{MeDI}{\mathbf{P}}_{\mathbf{C}}/\mathbf{Inpu}{\mathbf{t}}_{\mathbf{C}}\right) $$

The differential enrichment peaks were filtered according to the following criteria:

i). At least one of the two groups has a median (log2 MeDIP/Input) > =0.3 and M’> 0.
ii). At least half of probes in a peak may have coefficient of variability (CV) < = 0.8 in both groups.

Before hybridization to the array, genomic DNA was sonicated to random fragments in size of about 200–1000 bp. Immunoprecipitation of methylated DNA was performed using Biomag**™** magnetic beads coupled to a mouse monoclonal antibody against 5-methylcytidine. The immunoprecipitated DNA was eluted and purified by phenol chloroform extraction and ethanol precipitation. The total input and immunoprecipitated DNA were labeled with Cy3- and Cy5-labeled random 9-mers. Scanning was performed with the Axon GenePix 4000B microarray scanner. Raw data was extracted as pair files by NimbleScan software.

The pair files were analyzed with the tiling workflow in Partek Genomics Suite® version 6.6 (St. Louis, Missouri, USA). Nimblegen scan pair files (635 nm and 532 nm) for each sample were annotated against hg18 and enriched regions were detected using a two-way ANOVA between an affected twin and their unaffected co-twin. The enriched regions settings were set at a minimum p-value of 0.001 and the number of probes to call a region was set at a minimum of 4. MAT scores were generated for each differentially methylated region. Overlapping genes were then identified as those RefSeq (2014-04-29 version) genes that were either within the gene or 5000 bp upstream or 3000 downstream of the gene. Differentially methylated regions (DMRs) in each affected twin were identified in relation to the pattern in the well twin. Also, the presence or absence of each DMR was assessed as familial or *de novo* based on their presence or absence in Mom and/or Dad.

The identified genes with significant changes in DNA methylation between twins discordant for schizophrenia (DMRs) were then analyzed using Ingenuity Pathway Analysis (Ingenuity Systems Inc, CA, USA) towards identification of networks and canonical pathways overrepresented in the enriched genes. Also, pathway analysis and gene ontology analysis were conducted using Partek Pathways (Fishers Exact Test) and Enrichr [25]. Shared genes were annotated with imprinting data from GeneImprint (http://www.geneimprint.com) and The Catalogue of Parent of Origin Effects (http://igc.otago.ac.nz/home.html).

## Results

We report the genome-wide analysis of methylation differences in two families with monozygotic twins discordant for schizophrenia using the NimbleGen Human DNA Methylation Promoter Plus CpG Island 720k Array. The data was analyzed with Partek Genomics Suite and yielded three main lines of results presented below:

### MZ twins show differences in DNA Methylation profiles

**Figure 2:**
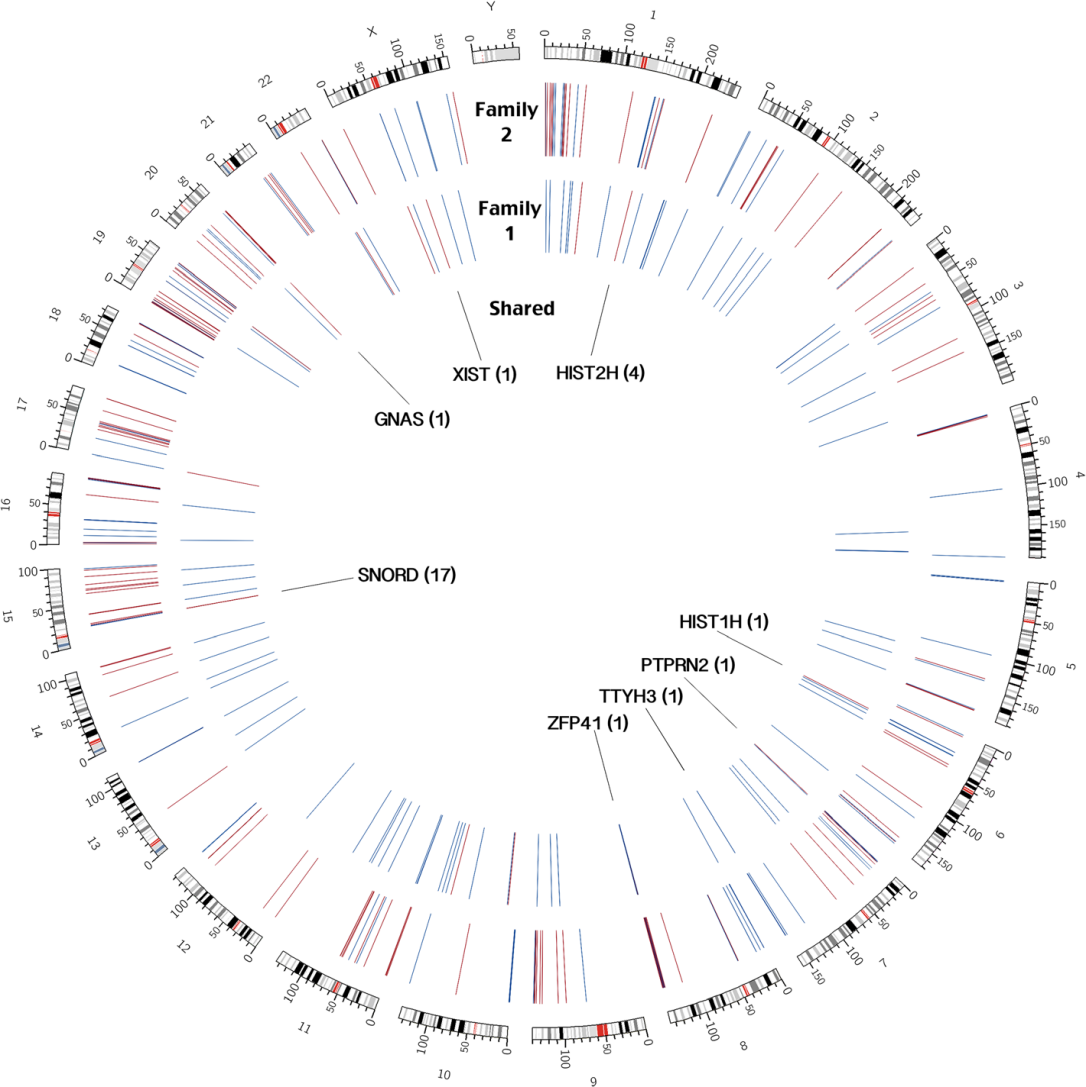
Differential methylation in two twin pairs in a Circos plot covering all chromosomes. Circos plot representing differentially methylated regions in each affected twin as well as shared regions between both affected twins in our study. Red represents a decrease in methylation in the affected twin and blue represents an increase in methylation in the affected twin. The outside track represents differentially methylated regions in the affected member of Family 2, the middle track represents differentially methylated regions in the affected member of Family 1 and the inside track represents the shared regions found in both unrelated affected twins. Affected genes are labeled.

**Figure 3:**
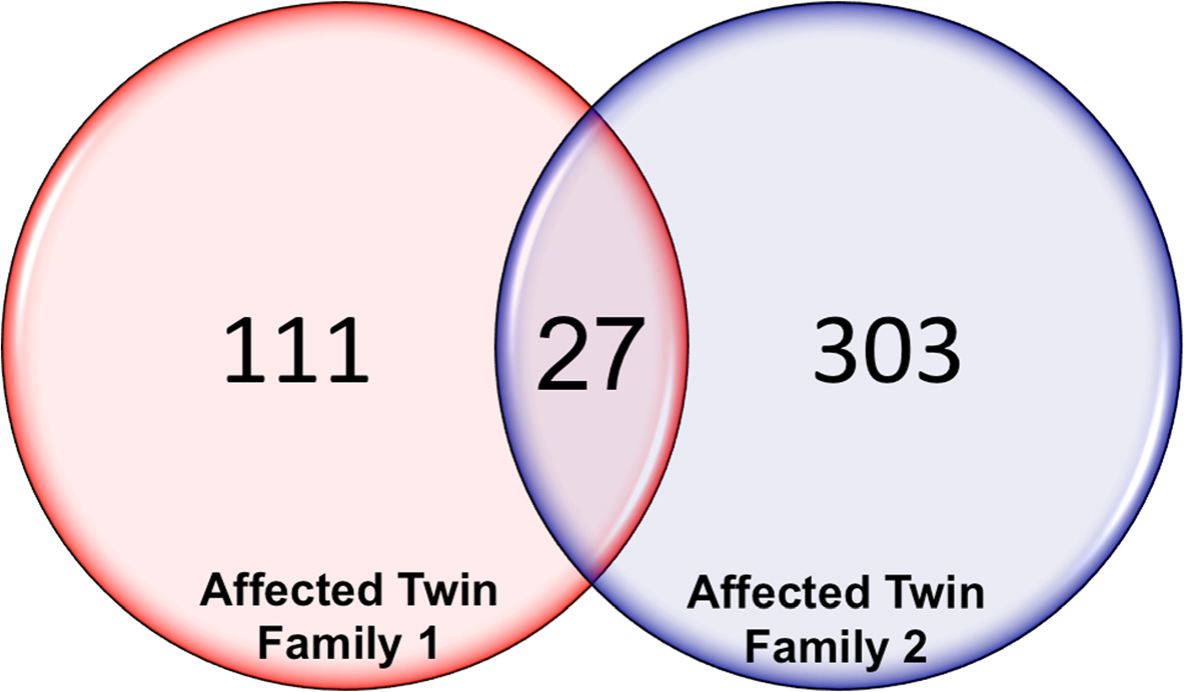
Overlap of differentially methylation regions. Venn diagram showing the number of genes differentially methylated in each patient (138 and 330 respectively) as well as the genes enriched in both affected twins in the study (27).

The genome-wide DNA methylation profiles have revealed differentially methylated peaks and regions (DMRs) between the MZ twin pairs in our study (p ≤ 0.001). Further, the availability of parental data has allowed us to assess each DMR for its presence/absence in the two parents. The results show that methylation profiles in twins include both shared and *de novo* events (arising from environmental exposures or random events). We note that in Family 1 as well as in Family 2 (Figure 1), most of the DMRs appear *de novo* (are not found in Mom or Dad) as only 25% and 13% of the DMRs, respectively, were present in either Mom or Dad. The results have also allowed identification of specific genes that are differentially methylated between the affected twin and their identical unaffected twin. Specifically, we note that 138 genes are differentially methylated in the twin pair from Family 1 (see Additional file 1) and 330 genes are differentially methylated in Family 2 (see Additional file 2). A visual representation of these results is given in Figure 2, specifically the outside track of the Circos diagram represents DMRs in Family 2, the middle track represents DMRs in Family 1 and the inside track represents 27 overlapping DMRs annotated with gene identity. An overlap between the DMRs present in the affected member of the two unrelated families (Figure 3) suggests that most (80-92%) of the DMRs are twin pair specific. Chromosome 1 (19 and 36 DMRs respectively) and Chromosome 15 (21 and 30 DMRs respectively) show the most DMRs in Family 1 as well as in Family 2. Family 2 also has a large number of DMRs on Chromosome 19 (30 DMRs).

### MZ twins discordant for schizophrenia share genomic regions of differential methylation

Figure 3 shows 27 genes that were differentially methylated in two affected twins. Of the genes that showed methylation differences in both sets of twins, 24 were increased in methylation status in the affected twins. The list of genes (Table 1) identified shared common regions with exact DMR start/end locations in the two patients. The exception was the *PTPRN2* (Entrez Gene: 5799), *TTYH3* (Entrez Gene: 80727) and *ZFP41* (Entrez Gene: 286128) regions where the DMRs were found to be nearby (Table 1). Also, the sequences affected are specific to the promoter regions as expected.

**Table 1:** **Differentially methylated regions identified in two affected MZD twins belonging to two unrelated families**

**Transcript**	**Chr**	**Region start**	**Region end**	**Distance to TSS**	**Methylation status**	**MAT score Family 1**	**MAT score Family 2**	**Found in Family 1 parental**	**Found in Family 2 parental**
HIST2H2AA3	1	148085850	148085870	−3382	Increase	8.45215	12.7557	YES (Both)	YES (Mom)
HIST2H2AA4	1	148085850	148085870	−3382	Increase	8.45215	12.7557	YES (Both)	YES (Mom)
HIST2H3A	1	148085850	148085870	−4935	Increase	8.45215	12.7557	YES (Both)	YES (Mom)
HIST2H3C	1	148085850	148085870	−4935	Increase	8.45215	12.7557	YES (Both)	YES (Mom)
HIST1H1C	6	26164302	26164322	357	Increase	6.0215	7.54892	NO	NO
PTPRN2	7	F1:157141154 F2:157352628	F1:157141174 F2:157352648	F1:932070 F2:720596	Increase	2.41616	7.11519	NO	NO
TTYH3	7	F1:2653547 F2:2664585	F1:2653567 F2:2664605	F1:15418 F2:26456	F1:Increase F2:Decrease	2.47725	−2.82779	NO	NO
ZFP41	8	F1:144403919 F2:144409425	F1:144403939 F2:144409445	F1:3446 F2: 8952	Increase	2.05379	15.1625	NO	NO
SNORD115-10	15	22983806	22983826	30	Increase	7.8983	7.49051	YES (Both)	YES (Both)
SNORD115-11	15	22983806	22983826	−1828	Increase	7.8983	7.49051	YES (Both)	YES (Both)
SNORD115-12	15	22983806	22983826	1935	Increase	7.8983	7.49051	YES (Both)	YES (Both)
SNORD115-29	15	22983806	22983826	−1828	Increase	7.8983	7.49051	YES (Both)	YES (Both)
SNORD115-33	15	23030052	23030072	2974	Increase	7.8983	7.49051	YES (Both)	YES (Both)
SNORD115-34	15	23030052	23030072	1425	Increase	7.8983	7.49051	YES (Both)	YES (Both)
SNORD115-35	15	23030052	23030072	−415	Increase	7.8983	7.49051	YES (Both)	YES (Both)
SNORD115-36	15	22983806	22983826	−1828	Increase	7.8983	7.49051	YES (Both)	YES (Both)
SNORD115-37	15	23030052	23030072	−4154	Increase	7.8983	7.49051	YES (Both)	YES (Both)
SNORD115-43	15	22983806	22983826	−1828	Increase	7.8983	7.49051	YES (Both)	YES (Both)
SNORD115-5	15	22983806	22983826	1932	Increase	7.8983	7.49051	YES (Both)	YES (Both)
SNORD115-9	15	22983806	22983826	1935	Increase	7.8983	7.49051	YES (Both)	YES (Both)
SNORD116-10	15	22868070	22868090	−2263	Increase	7.8983	7.49051	YES (Both)	YES (Both)
SNORD116-11	15	22868070	22868090	−4078	Increase	7.8983	7.49051	YES (Both)	YES (Both)
SNORD116-3	15	22868070	22868090	−1256	Increase	7.8983	7.49051	YES (Both)	YES (Both)
SNORD116-8	15	22868070	22868090	1399	Increase	7.8983	7.49051	YES (Both)	YES (Both)
SNORD116-9	15	22868070	22868090	−1256	Increase	7.8983	7.49051	YES (Both)	YES (Both)
GNAS	20	56879799	56879819	18368	F1:Decrease F2:Increase	−10.7502	17.2187	YES (Both)	YES (Both)
XIST	X	72974756	72974776	14538	F1:Decrease F2:Increase	−7.36623	12.1903	YES (Both)	NO

Chr = Chromosome, Region Start and Region End = Beginning and end of region, TSS = Distance to Transcript Start Site; F1 = Family 1; F2 = Family 2.

**Figure 4:**
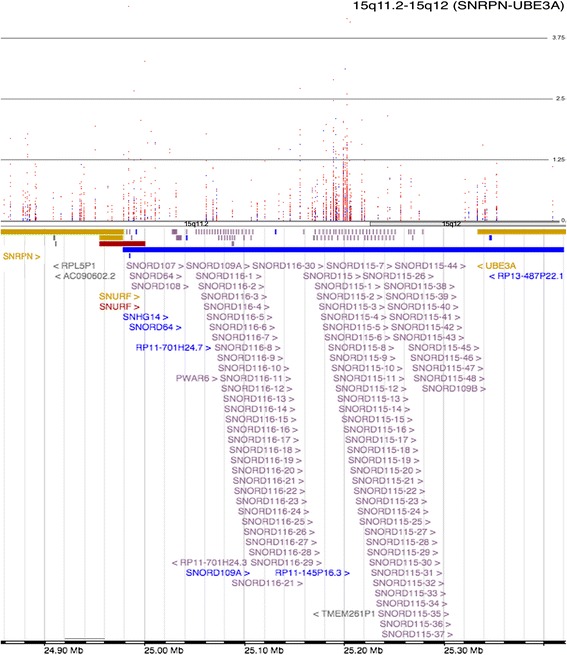
Manhattan plot representing methylation in the 15q11.2-15q12 region in the twin pairs of Family 1. This region spans from *SNRPN* to *UBE3A* and encompasses members of the *SNORD115* and *SNORD116* gene families (top). A red dot indicates a decrease in methylation in the affected twin. A blue dot indicates an increase in methylation in the affected twin (Family 1). This region contains a complex regulatory ncRNA involved in imprinting control and neurodevelopment.

Interestingly, a subset of common genes identified (5 genes), belong to either the *HIST2H* cluster on Chromosome 1 or the *HIST1H* region on Chromosome 6 (Table 1). Further, 17 of the 27 genes belong to either the *SNORD115* (Entrez Gene: 692218) or *SNORD116* (Entrez Gene: 692236) clusters on chromosome 15. All seventeen of the *SNORD* genes identified in two patients are known to be genomically imprinted and produce ncRNA transcripts that undergo extensive processing and form many functional classes of non-coding regulatory RNA (Table 1). This region can be seen in detail in Figure 4 that extends from the *SNRPN* gene (Entrez Gene: 6638) to the *UBE3A* gene (Entrez Gene: 7337) and encompasses the *SNORD115* and *SNORD116* gene families. Ingenuity Pathway Analysis (IPA) involving the shared genes has identified Protein Kinase A Signaling as the most enriched canonical pathway (*p* = 3.09E-04). In addition, Granzyme A Signaling (*p* = 6.83E-03), G Protein Signaling Mediated by Tubby (*p* = 1.24E-02), Serotonin Receptor Signaling (*p* = 1.72E-02) and UVB-Induced MAPK Signaling (*p* = 2.12E-02) were identified as canonical pathways of interest (Table 2c). IPA also identified *DRD4* (Entrez Gene: 1815), a dopamine receptor gene, to be the top upstream regulator of the twenty-seven common genes. Similarly, IPA identified developmental disorders (*p* = 4.03E-04-1.21E-03) as a top disease associated with this gene set while, Cell Signaling (*p* = 4.03E-04-3.73E-02), Nucleic Acid Metabolism (*p* = 4.03E-04-3.73E-02) and gene expression (*p* = 3.62E-03-9.63E-03) have been revealed as significant molecular and cellular functions. Also, Nervous System Development and Function (*p* = 1.61E-03-1.61E-03) has been revealed as one of the top physiological systems related to this gene set. Further, Infectious Disease, Hereditary Disorders, Embryonic Development and Cell Death and Survival were notable associated network functions related to the differentially methylated gene set in both schizophrenic twins. When the 27 genes were analyzed using Enrichr [25], expression in whole brain was identified as the top human gene atlas finding. Enrichr also identified OMIM disease classifications related to neurodevelopment to be enriched in our gene list; these include Asperger’s syndrome (*p* = 0.039) and mental retardation (*p* = 0.065).

**Table 2:** **Ingenuity pathway analysis (IPA) results a) Family 1 b) Family 2 c) Subset of 27 genes found in both affected twins**

**a. Family 1**		
**Biological function**	***P-value***	**Genes**
**Diseases and disorders**		
Developmental Disorder	3.93E-03 - 3.21E-02	10
Hereditary Disorder	3.93E-03 - 4.58E-02	19
Skeletal and Muscular Disorders	3.93E-03 - 3.21E-02	5
Neurological Disease	4.77E-03 - 3.54E-02	13
**Molecular and cellular functions**		
Carbohydrate Metabolism	7.88E-04 - 2.06E-02	3
Lipid Metabolism	7.88E-04 - 2.06E-02	3
Small Molecule Biochemistry	7.88E-04 - 4.58E-02	7
Gene Expression	2.63E-03 - 4.58E-02	18
Cell Death and Survival	5.20E-03 - 3.16E-02	13
**Canonical pathways**		
Human Embryonic Stem Cell Pluripotency	6.45E-05	6/149 (0.04)
Tec Kinase Signaling	1.51E-04	6/175 (0.034)
Sphingosine-1-phosphate Signaling	2.42E-03	4/115 (0.035)
Renal Cell Carcinoma Signaling	5.55E-03	3/71 (0.042)
IL-4 Signaling	6.49E-03	3/75 (0.04)
**Physiological system development and function**		
Cardiovascular System Development and Function	5.20E-03 - 3.58E-02	1
Hair and Skin Development and Function	5.20E-03 - 4.58E-02	2
Hematological System Development and Function	5.20E-03 - 4.13E-02	7
Immune Cell Trafficking	5.20E-03 - 4.13E-02	3
**Associated network functions**		
Cell Death and Survival, Cellular Movement, Cellular Function and Maintenance		Score = 19
Cellular Movement, Immune Cell Trafficking, Hematological System Development and Function		Score = 17
Hereditary Disorder, Skeletal and Muscular Disorders, Developmental Disorder		Score = 9
Connective Tissue Disorders, Dermatological Diseases and Conditions, Hematological System Development and Function		Score = 2
Organ Morphology, Reproductive System Development and Function, Cellular Function and Maintenance		Score = 2
**b. Family 2**		
Cancer	2.09E-04 - 4.07E-02	204
Gastrointestinal Disease	8.86E-04 - 4.07E-02	29
Organismal Injury and Abnormalities	9.34E-04 - 3.94E-02	46
Skeletal and Muscular Disorders	9.34E-04 - 4.07E-02	36
**Molecular and cellular functions**		
Lipid Metabolism	1.88E-04 - 4.07E-02	7
Molecular Transport	1.88E-04 - 4.07E-02	9
Small Molecule Biochemistry	1.88E-04 - 4.07E-02	19
Cellular Growth and Proliferation	3.06E-04 - 4.07E-02	21
Cell Morphology	5.59E-04 - 3.63E-02	10
**Canonical pathways**		
Hepatic Cholestasis	1.05E-04	9/141(0.064)
Granzyme A Signaling	1.51E-03	3/17 (0.176)
Ovarian Cancer Signaling	2.24E-03	7/138 (0.051)
STAT3 Pathway	3.31E-03	5/74 (0.068)
Colorectal Cancer Metastasis Signaling	4.9E-03	9/244 (0.037)
**Physiological system development and function**		
Cardiovascular System Development and Function	1.88E-04 - 4.07E-02	10
Digestive System Development and Function	1.88E-04 - 1.88E-04	2
Embryonic Development	1.88E-04 - 4.07E-02	14
Organ Development	1.88E-04 - 4.07E-02	6
**Associated network functions**		
Cell Death and Survival, Cellular Development, Connective Tissue Development and Function		Score = 39
Cellular Movement, Immune Cell Trafficking, Hematological System Development and Function		Score = 12
Cancer, Organismal Injury and Abnormalities, Reproductive System Disease		Score = 11
Cell Death and Survival, Cellular Movement, Renal Necrosis/Cell Death		Score = 11
Cell Morphology, Cellular Function and Maintenance, Cell Cycle		Score = 9
**c. Shared DMRs between both families**		
Cancer	4.03E-04 - 4.50E-02	5
Connective Tissue Disorders	4.03E-04 - 8.05E-04	1
Developmental Disorder	4.03E-04 - 1.21E-03	1
Endocrine System Disorders	4.03E-04 - 4.50E-02	2
**Molecular and cellular functions**		
Cell Signaling	4.03E-04 - 3.73E-02	1
Nucleic Acid Metabolism	4.03E-04 - 3.73E-02	1
Small Molecule Biochemistry	4.03E-04 - 3.73E-02	1
Gene Expression	3.62E-03 - 9.63E-03	1
Cellular Movement	6.43E-03 - 6.43E-03	1
**Canonical pathways**		
Protein Kinase A Signaling	3.09E-04	3/368 (0.008)
Granzyme A Signaling	6.83E-03	1/17 (0.059)
G Protein Signaling Mediated by Tubby	1.24E-02	1/31 (0.032)
Serotonin Receptor Signaling	1.72E-02	1/43 (0.023)
UVB-Induced MAPK Signaling	2.12E-02	1/53 (0.019)
**Physiological system development and function**		
Nervous System Development and Function	1.61E-03 - 1.61E-03	1
Hematological System Development and Function	6.43E-03 - 2.47E-02	1
Immune Cell Trafficking	6.43E-03 - 6.43E-03	1
Behavior	2.04E-02 - 2.04E-02	1
**Associated network functions**		
Infectious Disease, Cancer, Gastrointestinal Disease		Score = 3
Tissue Morphology, Organismal Survival, Gene Expression		Score = 3
Hereditary Disorder, Gene Expression, Embryonic Development		Score = 3
DNA Replication, Recombination, and Repair, Gene Expression, Cancer		Score = 3
Cell Death and Survival, Cellular Function and Maintenance, Connective Tissue Development and Function		Score = 3

### Unrelated MZ twins discordant for schizophrenia have differentially methylated networks; some pair specific and others shared

IPA analysis on the DMRs identified in Family 1 shows that they are implicated in Developmental Disorders (*p* = 3.93E-03-3.21E-02), Hereditary Disorders (*p* = 3.93E-03-4.58E-02), Skeletal and Muscular Disorders, (*p* = 3.93E-03-3.21E-02) and Neurological Disease (*p* = 4.77E-03-3.54E-02) as these were the top diseases and disorders associated with the DMR gene set (Table 2). Similarly, in Family 2, they are implicated in Skeletal and Muscular Disorders (*p* = 9.34E-04-4.07E-02) as a top disease and disorder followed by Cancer (*p* = 2.09E-04-4.07E-02), Gastrointestinal Disease (*p* = 8.86E-04-4.07E-02) and Organismal Injury and Abnormalities (p = 9.34E-04-3.94E-02). This analysis also identified a number of interesting canonical pathways including Human Embryonic Stem Cell Pluripotency (*p* = 6.45E-05), Tec Kinase Signaling (*p* = 1.51E-04) and IL-4 Signaling (*p* = 6.49E-03) in Family 1. In contrast, the canonical pathways of interest in Family 2 included Hepatic Cholestasis (*p* = 1.05E-04), Granzyme A Signaling (*p* = 1.51E-03) and STAT3 Pathway (*p* = 3.31E-03). Other twin specific networks and functions identified are listed in Tables 2a and b.

**Figure 5:**
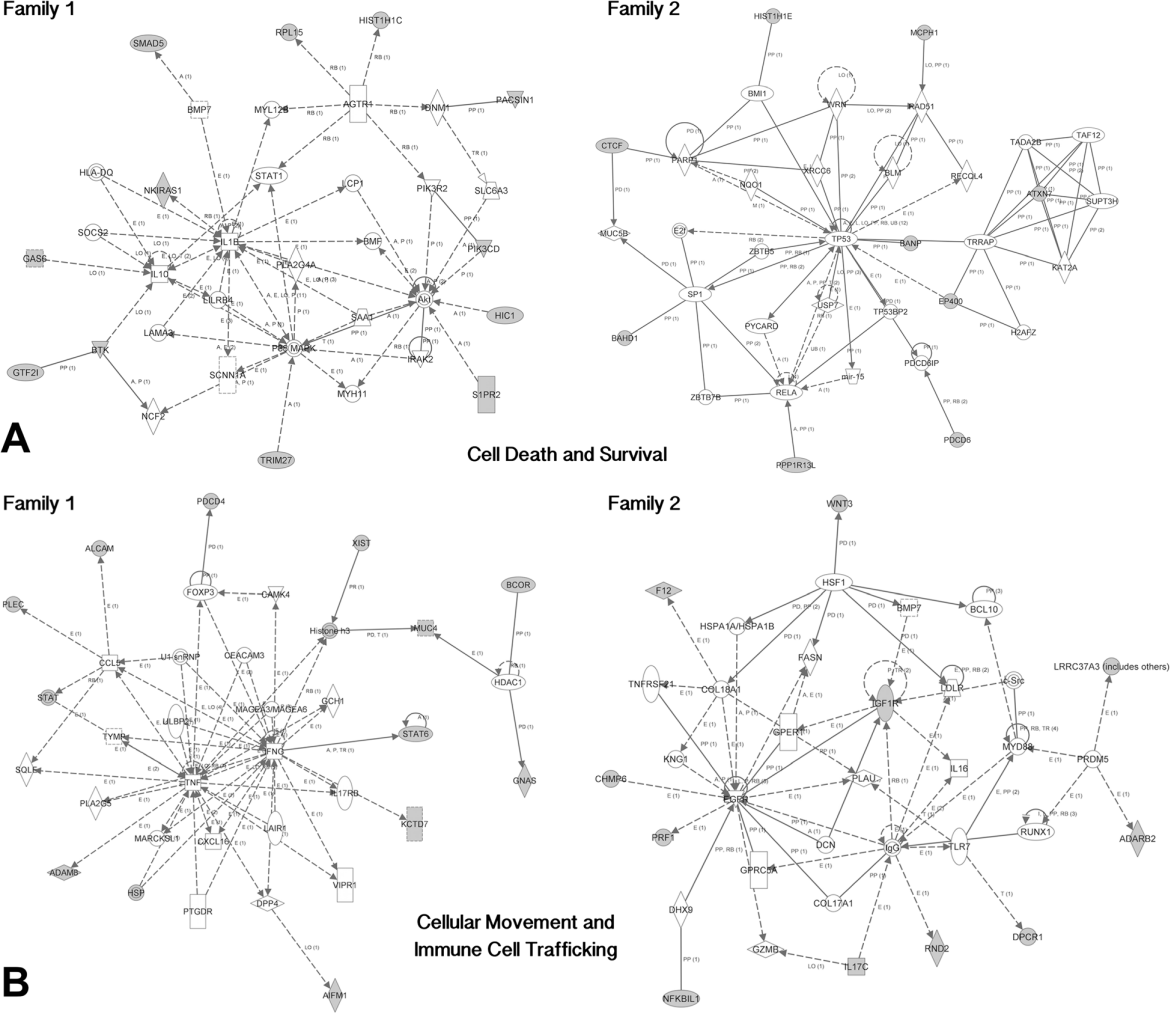
Common networks identified in both families. **a.** Two cell death and survival networks independently identified in each affected twin in our study. Ingenuity Pathway Analysis (IPA) was used to identify the networks in both affected twins in our study. Shading represents genes in our study that are differentially methylated in the network. **b.** Two cellular movement and immune cell trafficking networks independently identified in each affected twin in our study. Ingenuity Pathway Analysis (IPA) was used to identify the networks in both affected twins in our study. Shading represents genes in our study that are differentially methylated in the network.

This analysis also identified two functional networks that were affected in both unrelated twin pairs (Figure 5). They included a cell death and survival network (ratio of differentially methylated genes to total number of genes in the network was 12/35 and 9/35, in Family 1 and Family 2, respectively) (Figure 5a) and a cellular movement and immune cell trafficking network (14/35 and 11/35 genes in Family 1 and 2, respectively) (Figure 5b). In Family 1, the Cell Death and Survival network identified *IL1B* (Entrez Gene: 3553) as the primary hub gene of the network, while in Family 2, *TP53* (Entrez Gene: 7157) was identified as the primary hub gene in this network. Similarly, the cellular movement and immune cell trafficking networks involves *TNF* (Entrez Gene: 7124) and *IFNG* (Entrez Gene: 3458) as hub genes in Family 1 and *IGF1R* (Entrez Gene: 3480) and *EGFR* (Entrez Gene: 1956) as hub genes in Family 2. The results may argue that networks rather than genes may ultimately underlie the etiology of schizophrenia and related disorders.

## Discussion

The results included in this report support that monozygotic twins differ in DNA methylation. This difference is genome-wide and includes a relatively large number of presumed *de novo* events. The results suggest that *de novo* methylation changes are common during development and aging in the two pairs studied. The results therefore are specific to the two patients and will not necessarily cover the whole spectrum of the disease. Taken together, the results call for further assessment of epigenetic profiles in an increased number of rare discordant twin pairs, as the analysis included in this report is based on two female pairs only. The DMRs may represent random events over the lifetime or arise from shared environmental conditions [20,21,26]. Further, although the two MZD pairs for schizophrenia are unrelated, they share differences in DNA methylation in 27 genes and genomic locations. Interestingly, many of these shared differences belong to the histone coding gene family, which has already been implicated in the causation of schizophrenia [12,21,27]. Also, the differentially methylated genes affect two networks that are compatible with the development of this neurodevelopmental disease. Finally, the genes identified have the potential to explain the discordance of the two twin pairs for schizophrenia. Of special interest to this discussion are *HIST* genes primarily located on chromosome 1 and *SNORD115* and *SNORD116* genes located on chromosome 15. In addition, the two patients share identified networks affecting cell death and immune cell trafficking, which are elaborated on below.

It is noteworthy that our study identified 5 *HIST* genes as genes of interest in the discordance for schizophrenia. A Histone gene cluster on Chromosome 6 has been previously implicated in a meta-analysis of schizophrenia associated loci in individuals of European ancestry [28]. The implicated region itself, 6p22.1, was also found to be associated with schizophrenia [28]. In addition, histone methylation has been found in olfactory cells implicating oxidative stress in schizophrenia [29]. Lastly, postmortem brain tissue from schizophrenia patients has been found to have higher levels of histone deacetylase, *HDAC1*, and the level of *HDAC1* has been shown to be inversely correlated with *GAD67* (Entrez Gene: 2571) protein expression, which tends to be decreased in schizophrenia patients [30,31] and argues that histones may play a role in this complex disease.

The *SNPRN-UBE3A* locus, which encompasses the *SNORD115* and *SNORD116* gene families, is a complex non-coding RNA region that spans 15q11-q13 [32]. Noncoding RNAs, including miRNAs, are known to fine-tune gene expression through transcriptional and post-transcriptional regulations including RNA stability and protein translation [33]. In addition to serving as an antisense RNA for *UBE3A*, the polycistronic transcript is also a host that undergoes extensive processing, including the production of a number of small nucleolar RNA species (snoRNAs). The HBII-52 snoRNAs (also known as the *SNORD115* family) regulate the alternative splicing of the 5HTR2C serotonin receptor and result in an increased serotonin response in neurons [34]. *SNORD115* is further processed into processed snoRNAs (psnoRNAs) that go on to regulate alternative splicing in a number of other transcripts, including epigenomic modifiers [35]. However, a conflicting report emerged in 2012 that showed evidence against psnoRNAs, indicating that *SNORD115* and *SNORD116* may generate genuine snoRNAs [36]. A novel ncRNA species, LncRNAs with snoRNA ends, also originates from this loci. They are functionally distinct from snoRNAs and lncRNAs and are associated with the FOX family of splicing regulators that alter the alternative splicing of a number of other genes. In addition to psnoRNAs, snoRNAs, and lnc-snoRNAs, the snoRNAs are even further processed into snoRNA-derived RNAs (sdRNAs). These sdRNAs are proposed to come in two variations: some resembling miRNAs that associate with argonaute proteins to regulate translation and another longer type that form complexes to influence gene expression [37]. Further investigation into the locus has shown that it produces even more ncRNA products, with the introns forming the snoRNA derivatives and the exons forming two distinct but overlapping neuronal lncRNA clouds from the *SNORD 115* and *SNORD116* regions that are involved in modulating circadian rhythm and energy expenditure [38,39]. The lncRNAs are functionally distinct from the earlier identified ncRNA species and are also primarily expressed in developing neurons.

Interestingly, the lncRNA from the *SNPRN-UBE3A* region has been suggested to regulate another imprinted locus, the *DLK1-DIO3* region, which is the only other known imprinted cluster of ncRNA that produces lncRNA, miRNA, and snoRNA. It is also involved in neurodevelopment and suggests that imprinted ncRNAs are capable of ‘genomic cross-talk’ [40,41]. Interestingly, while imprinting disorders are known to originate from these loci, a highly resolved and restricted deletion in the *SNORD116* region was identified as the minimal mutation to cause Prader-Willi Syndrome [42]. Also, *DRD4*, a top upstream regulator identified in the shared *DMR* gene set, has been previously implicated in schizophrenia, and is thought to be the target of many antipsychotics [43].

The identified networks across unrelated twins share common functions supporting the hypothesis that a different set of patient specific gene insults may lead to disease symptoms. There has been a long held linkage between schizophrenia and immune cell function. This theory gained further support as novel functions of immune molecules in the brain and cross-talk between the immune system and the central nervous system [44]. In addition, a number of studies have shown up-regulation of immune-inflammatory genes in the CNS [44-46] as well as immune system gene modulation of synaptic function [47]. In the cellular movement and immune cell trafficking networks identified in Family 1, two genes (*TNF* and *IFNG)* immerged as hub genes. The tumor necrosis factor (*TNF*α) had been associated with schizophrenia and also it was reported that immune dysregulation could have a genetic component in schizophrenia patients [48]. Also, a single nucleotide polymorphism in the interferon gamma gene (*IFNG*) had been associated with paranoid schizophrenia in males [49], however, the role of the gene in the pathophysiology of the disease remains to be elucidated. Similarly, the other hub gene of cellular movement and immune cell trafficking network, *EGFR,* identified in Family 2 have also been associated with schizophrenia [50].

The primary hub gene in the Cell Death and Survival network, *IL1B*, which is differentially methylated in Family 1, has an important role in the development of the central nervous system. Also, it is reported to be associated with schizophrenia [51]. Similarly, the human *p53* tumor suppressor gene (*TP53*), which is identified as a primary hub gene in the cell death and survival network of Family 2 in this study, plays a role in neurodevelopment and was previously associated with schizophrenia [52].

Finally, the question of the effect of the observed DNA methylation on disease must be interpreted with caution. We may add that most schizophrenia patients are exposed to antipsychotic drugs in North America. Our patients were under treatment with medications, though not consistently. We note that such drugs and cellular heterogeneity of the studied samples [53] may also affect DNA methylation [54] as confounding factors. Further, although practical, we recognize the use of blood to make inferences regarding a brain-based disorder is not without caution and recommend that this discovery study be complemented by population studies of larger sample size on this disease as well as confirmation of the findings using alternative technologies. However, in its current state, it is not possible to eliminate these and other confounding factors that may affect our results at this time. The specific observations on genes and pathways relevant to the disease however, lend support to the complexity of this neurodevelopmental disease and its aetiology.

## Conclusions

We report genome-wide methylation differences between monozygotic twins discordant for schizophrenia. A number of genes and networks identified are twin pair-specific, while others are shared between unrelated patients. Most patient specific genes and gene networks affected have been previously implicated in schizophrenia. Specifically, the results identify three sets of gene clusters, *HIST* (Chromosome 1), *SNORD115* and *SNORD116* (Chromosome 15), which are differentially methylated in the twins with schizophrenia as compared to their unaffected counterpart. We also report common networks identified independently in the two patients that relate to cell death/survival and immune cell trafficking networks disrupted in schizophrenia. The results on monozygotic discordant twins argue for a network based rather than gene focused approach in the understanding of schizophrenia and related disorders.

### Availability of supporting data

The data set supporting the results of this article is available in the Gene Expression Omnibus (GEO) repository, [GSE61862, http://www.ncbi.nlm.nih.gov/geo/query/acc.cgi?acc=GSE61862].

## Additional files

Additional file 1:
**Genes differentially methylated between co-twins in Family 1.** The regions represent 138 unique genes. Additional file 2:
**Genes differentially methylated between co-twins in Family 2.** The regions represent 330 unique genes.

## Abbreviations

DMR: Differentially methylated region
IPA: Ingenuity pathway analysis
MeDIP: Methylated DNA immunoprecipitation
MZ: Monozygotic
MZD: Discordant monozygotic
psnoRNA: Processed snoRNA
sdRNA: snoRNA-Derived RNA
snoRNA: Small nucleolar RNA species
